# Compound Fracture and Dislocation of the Astragalus

**Published:** 1849-01

**Authors:** 


### SURGERY.

*Compound Fracture and Dislocation of the Astragalus.*—Mr. Faircloth detailed the following case of this accident:

Ephraim Scott, a lad in a racing stable at Newmarket, of ordinary stature, quiet disposition, with brown hair and eyes, aged fourteen years, had always enjoyed good health, and had had no serious illness except measles. Saturday, May 3d, was riding a young and vicious horse in the paddock; the animal reared until it overbalanced itself, and fell back, rolling over on its right side. The boy came down on his right side, shoulder and hip; his right leg extended forward, received the horse's neck across the knee, whilst the left still entangled in the stirrup, projected backwards and under the horses right flank and hip. Except as regarded this limb, he escaped with a few trifling bruises, but the left ankle presented the following appearance:—Before the shoe-lace and the stocking were removed, a large projection on the outer side of the joint and some arterial oozing; when uncovered, the bones were seen projecting through a semilunar wound, at least four inches long. The astragalus was dislocated from its connection with the os calcis, and this articulating surface turned outwards through the lips of the wound. The bone was not separated from its attachments to the tibia and fibula, nor from the scaphoid anteriorly, but to allow the bone to turn half round as above described, the neck had given way transversely. Both the anterior portion of bone, which remained *in situ*, attached to the scaphoid, and the other large portion, were chipped or broken more or less.

*Treatment.*—After a very careful examination of the parts, the foot, the vessels, and the boy's state generally, it was resolved to try and save the limb. The dislocated portion of the astragalus, (amounting to four-fifths of the whole of that bone,) was carefully detached from the tibia and fibula and these bones let down upon the calcis. The edges of the wound were brought together with three or four stitches and plaster, a compress of lint was placed over them, covered with oiled silk, confined with a turn or two of the bandage, and the whole secured in Macintyre's splint. During the first week he suffered from irritative fever, but much less so than might have been anticipated. He took salines and aperients, &c., as required. There was very little oozing of blood after the first few hours, but synovia was discharged in considerable quantities. The treatment was persevered in, the wound being dressed still with dry lint under oiled silk, as often as necessary until about fourteen days, and the splint itself was of course re-applied. This was done continually until about seven weeks from the date of the accident, when it was discarded, and an inside wooden splint with a foot-piece substituted for it, and a bread poultice employed. At the expiration of another week this support was removed, the limb bandaged, (a pad of lint only, being used for dressing) and a stirrup of paste-board passed under the foot, and continued up either side of the leg. He now got up and walked about, aided with crutches, the foot being supported in a sling. *Seven* days after this time he began to try and accustom himself to bear slightly on the foot. He soon left off one crutch and used a stick. The wound was touched with sulphate of copper from time to time, and only covered with lint.

November 6th. He now walks with a crutch and stick, when going any distance, and walks well. The ankle is almost motionless as regards flexion and extension.

April 10th, 1848. Walks well with a stick, frequently without one; the limb is nearly an inch shorter than the opposite one. Flexion and extension movements of joint increase.—*Med. and Surg. Journal.*

